# Sodium-Glucose Cotransporter 2 (SGLT2) Inhibitors and Lipid Modulation in Heart Failure: A Narrative Review

**DOI:** 10.7759/cureus.99752

**Published:** 2025-12-21

**Authors:** Sina Neshat, Hazhir Moradi, Matin Bidares, Afshin Heidari, Alireza Falahati Marvast, Ronal Ortega, Kiyan Heshmat

**Affiliations:** 1 Department of Biostatistics and Epidemiology, University of California San Francisco, San Francisco, USA; 2 Nosocomial Infections Reasearch Center, Isfahan University of Medical Sciences, Isfahan, IRN; 3 Health Policy Research Center, Shiraz University of Medical Sciences, Shiraz, IRN; 4 School of Medicine, Isfahan University of Medical Sciences, Isfahan, IRN; 5 Department of Toxicology and Pharmacology, School of Pharmacy and Pharmaceutical Sciences, Shahid Sadoughi University of Medical Sciences, Yazd, IRN; 6 School of Medicine, Faculty of Health, University of Pamplona, Pamplona, COL; 7 Department of Cardiology, Heart Failure Research Center, Cardiovascular Research Institute, Isfahan University of Medical Sciences, Isfahan, IRN

**Keywords:** canagliflozin, dapagliflozin, empagliflozin, heart failure, lipid profiles, sglt-2 inhibitors

## Abstract

In heart failure (HF), atherogenic dyslipidemia and lipotoxicity contribute to adverse remodeling. Sodium-glucose cotransporter 2 inhibitors (SGLT2i) improve HF outcomes, yet their lipid effects remain debated. This review aims to synthesize quantitative changes in lipid parameters and plausible mechanisms by which SGLT2i modulate lipoproteins in HF. Across trials and HF-focused cohorts, SGLT2i are associated with small increases in low-density lipoprotein cholesterol (LDL-C) and high-density lipoprotein cholesterol (HDL-C) and small decreases in triglycerides. Beyond concentrations, emerging data suggest qualitative remodeling - a shift toward less atherogenic LDL phenotypes (small-dense LDL (sd-LDL)↓) and increases in HDL2 - although evidence is limited and heterogeneous. Mechanistically, enhanced adipose lipolysis and hepatic β-oxidation/ketogenesis may raise ketone availability for the myocardium ("thrifty substrate"), while hepatic cholesterol pool-driven LDL receptor (LDLR) downregulation could explain modest LDL-C increases. These lipid shifts coexist with consistent reductions in HF events, independent of diabetes, implying benefits not captured by traditional lipid metrics alone. In HF, SGLT2i likely exert modest quantitative lipid changes but potentially meaningful qualitative lipoprotein remodeling alongside improved metabolic flexibility. Clinically, apolipoprotein B (ApoB)-targeted therapy (e.g., statins ± ezetimibe) remains essential when LDL-C/ApoB are above goal, with SGLT2i used for cardiorenal benefit. HF-specific trials powered for ApoB, sd-LDL, low-density lipoprotein particle number (LDL-P), HDL function, and lipidomics are lacking. In conclusion, SGLT2i produce small, mixed lipid changes in HF, but mechanistic and particle-level effects may align with improved outcomes; definitive HF-centric lipid studies are a priority.

## Introduction and background

Heart failure (HF) remains a major global health challenge, with high morbidity and mortality despite advances in guideline-directed therapy. Beyond neurohormonal activation and hemodynamic stress, metabolic disturbances - including atherogenic dyslipidemia and myocardial lipotoxicity - are increasingly recognized as key contributors to HF progression and adverse outcomes [1,2]. Altered lipid metabolism in HF is not only associated but also mechanistically relevant. Elevated triglycerides promote excess delivery of fatty acids to cardiomyocytes, increasing mitochondrial reactive oxygen species (ROS) generation and driving oxidative stress. Small-dense low-density lipoprotein (sd-LDL) particles penetrate the vascular endothelium more readily, undergo rapid oxidation, and amplify inflammatory signaling pathways that contribute to microvascular dysfunction and adverse remodeling. Impaired high-density lipoprotein (HDL) function reduces cholesterol efflux capacity and antioxidant activity, limiting the myocardium’s ability to counteract lipid toxicity and vascular inflammation. Together, these abnormalities create a pro-oxidative, pro-inflammatory, and metabolically rigid environment that accelerates maladaptive ventricular remodeling in HF [2].

Sodium-glucose cotransporter 2 inhibitors (SGLT2i), initially developed as antihyperglycemic agents, have now become foundational therapy for HF across the ejection-fraction spectrum, independent of diabetes status. Landmark outcome trials such as EMPA-REG OUTCOME, DAPA-HF, and EMPEROR-Reduced demonstrated substantial reductions in HF hospitalization and cardiovascular mortality with empagliflozin and dapagliflozin [3-5]. These benefits have been reproduced in patients with preserved ejection fraction (EF) [6,7] and individuals without diabetes, supporting broad cardioprotective effects beyond glucose lowering. Notably, available data do not show major phenotype-specific differences in lipid responses to SGLT2 inhibitors, although small observational studies suggest slightly greater triglyceride and small-dense LDL improvements among patients with obesity, insulin resistance, or type 2 diabetes. However, HF-specific, lipid-stratified analyses across EF phenotypes remain limited.

The mechanisms underpinning these benefits extend beyond natriuresis and hemodynamic unloading to include metabolic effects such as enhanced adipose lipolysis, accelerated hepatic β-oxidation, increased ketone utilization, and reduced accumulation of toxic lipid intermediates [8-11]. These adaptations suggest that modulation of lipid handling may represent a biologically relevant pathway in HF.

However, the effect of SGLT2i on lipid profiles remains an area of active debate. Meta-analyses and HF-focused cohorts report small increases in LDL-C and HDL-C with modest reductions in triglycerides [12-14]. Importantly, emerging data suggest qualitative improvements in lipoprotein composition, including reductions in small-dense LDL and increases in large-buoyant HDL subfractions, particularly with dapagliflozin and empagliflozin [12,15]. These findings raise the possibility that SGLT2i-related lipid changes may reflect improved metabolic efficiency and lipoprotein remodeling rather than atherogenic harm.

Given the expanding use of SGLT2i in HF and the lack of HF-specific lipid-focused trials, this narrative review synthesizes current evidence on quantitative lipid changes, emerging particle-level alterations, and mechanistic pathways linking SGLT2 inhibition to lipid metabolism in HF. Knowledge gaps and priorities for future research are also highlighted (see Appendix 1).

Accordingly, this narrative review aims to synthesize the quantitative and qualitative lipid effects of SGLT2 inhibitors in patients with heart failure and to discuss their clinical implications, particularly as they relate to contemporary lipid-lowering strategies in this population.

## Review

Because this manuscript was designed as a narrative review, a formal systematic framework was not applied; however, to ensure transparency, the literature search process is outlined here. We searched PubMed/MEDLINE, Embase, and the Cochrane Library for English-language studies published from January 2010 through May 2024, with the last search performed on May 30, 2024. Core search terms included combinations of: “heart failure,” “HFrEF,” “HFpEF,” “SGLT2 inhibitor,” “empagliflozin,” “dapagliflozin,” “canagliflozin,” “ertugliflozin,” “lipids,” “triglycerides,” “ApoB,” “LDL-C,” “HDL-C,” “LDL particle number,” “small-dense LDL,” “sd-LDL,” and “HDL function.”

We prioritized randomized controlled trials, HF-focused cohorts, mechanistic studies, and translational investigations reporting changes in lipid parameters or lipoprotein composition with SGLT2 inhibitors. When HF-specific lipid data were limited, metabolic studies in type 2 diabetes or high-risk cardiometabolic populations were included selectively to contextualize mechanistic pathways, provided they reported relevant lipid or particle-level outcomes. Case reports and studies without lipid endpoints were excluded.

Mechanistic links between SGLT2 inhibition, lipid metabolism, and heart failure

Shift in Substrate Utilization and Lipid Oxidation

SGLT2 inhibition promotes a metabolic shift characterized by enhanced adipose lipolysis, increased hepatic β-oxidation, and greater ketone body production [8-11]. In heart failure, where myocardial metabolic flexibility is impaired, this "thrifty substrate" phenomenon allows the myocardium to utilize ketone bodies more efficiently than fatty acids, improving energy yield per unit of oxygen [9, 11]. Such metabolic remodeling may contribute to improved ventricular energetics, reduced oxidative stress, and attenuation of myocardial lipotoxicity in patients with HF [8-10].

The rise in circulating free fatty acids during SGLT2 inhibition increases hepatic acetyl coenzyme A (acetyl-CoA) flux, thereby facilitating both ketogenesis and cholesterol synthesis [10,16]. This cross-talk between lipid and carbohydrate metabolism can lead to small, clinically insignificant elevations in LDL-C. Importantly, the reduction in intracellular lipid-toxic intermediates such as diacylglycerol and ceramides helps mitigate insulin resistance and lipotoxic damage, indirectly improving systemic lipid handling [8-11].

These metabolic adaptations collectively form the biological foundation for the modest but mechanistically coherent lipid changes observed with SGLT2 inhibitors in HF (Table 1).

**Table 1:** Proposed Mechanisms Linking SGLT2 Inhibitors to Lipid Modulation in Heart Failure Key Point: SGLT2 inhibitors may deliver small numerical lipid changes but potentially favorable qualitative remodeling and enhanced metabolic efficiency in heart failure. Abbreviations: SGL2 = sodium-glucose cotransporter 2; LDL = low-density lipoprotein; HDL = high-density lipoprotein; HF = heart failure; RAAS = renin-angiotensin-aldosterone system; TG = triglycerides. References:  [8-11,16]

Mechanistic Pathway	Expected Lipid Effect	Heart Failure–Related Impact	Evidence Type
↑ Adipose lipolysis → hepatic β-oxidation → ketogenesis	↓ TG	Enhanced cardiac energy efficiency ("ketone-fuel shift")	Metabolic physiology & translational studies
↑ Hepatic cholesterol pool → ↓ LDL receptor expression	↑ LDL-C (modest)	Minor LDL-C rise without adverse HF signal	Mechanistic & animal data
↓ Lipotoxic intermediates (diacylglycerol, ceramides)	↓ small-dense LDL (hypothesis supported by limited data)	↓ myocardial lipotoxicity; improved insulin sensitivity	Experimental models & limited clinical cohorts
Natriuresis, ↓ RAAS & inflammation	Potential ↑ HDL functionality	Improved vascular & endothelial health	Clinical biomarkers & mechanistic rationale
Shift toward metabolic flexibility	Mixed mild lipid effects	Improved myocardial energetics, ↓ oxidative stress	HF trials + mechanistic reviews

Impact on Lipoprotein Clearance and ApoB Kinetics

Preclinical studies have demonstrated that SGLT2 inhibition can transiently suppress hepatic LDL receptor (LDLR) expression because of an increased intracellular cholesterol pool [16,17]. This mechanism plausibly explains the small LDL-C increases observed in clinical studies [12-14]. Despite these changes, major cardiovascular outcome trials such as EMPA-REG OUTCOME and CANVAS found no associated increase in ischemic events, suggesting that these effects are metabolically benign [5,18].

Human data regarding ApoB kinetics remain limited, but available evidence indicates either neutral or slight reductions in ApoB and non-HDL-C levels, particularly in individuals with improved insulin sensitivity [15]. Taken together, these findings imply that SGLT2i-induced lipid modifications may reflect improved substrate utilization and redistribution rather than atherogenic upregulation of lipoprotein synthesis.

Qualitative Lipoprotein Remodeling

Beyond quantitative lipid parameters, SGLT2 inhibitors appear to promote qualitative remodeling of lipoprotein subclasses. Multiple clinical studies have shown that empagliflozin and dapagliflozin reduce levels of small-dense LDL (sd-LDL) and increase HDL2, the large and buoyant HDL subfraction [12,15]. Since sd-LDL and dysfunctional HDL are closely linked to adverse cardiovascular outcomes, these compositional shifts likely represent an anti-atherogenic adaptation rather than harm.

Emerging metabolomic data further support this interpretation, indicating enhanced lipid oxidation and reduced lipid peroxidation markers during SGLT2i therapy [15,16]. The overall direction of these findings suggests a favorable lipoprotein profile and improved metabolic efficiency, especially in the context of HF, where lipotoxicity and impaired lipid clearance are central pathophysiologic features.

Quantitative effects of SGLT2 inhibitors on lipid parameters in heart failure

Across randomized clinical trials and HF-focused cohorts, SGLT2 inhibitors have demonstrated modest and consistent changes in serum lipids, typically consisting of small increases in LDL-C and HDL-C and mild reductions in triglycerides [12-14]. The general pattern of lipid change among different agents is summarized in Table 2.

**Table 2:** Summary of Lipid Changes with SGLT2 Inhibitors in Heart Failure Patients Key Interpretation: Changes are generally modest in magnitude. Potential clinical relevance may lie more in lipoprotein quality shifts (lower small-dense LDL, higher HDL2) and metabolic effects rather than absolute LDL-C changes. Abbreviations: LDL-C = low-density lipoprotein cholesterol; HDL-C = high-density lipoprotein cholesterol; TG = triglycerides; ApoB = apolipoprotein B; HDL2 = large buoyant HDL fraction. References: [12-30]

Drug (Typical dose)	LDL-C	HDL-C	Triglycerides	Lipoprotein Quality / Subfractions	Heart Failure–Specific Comment
Empagliflozin (10–25 mg)	↑ slight (≈ +2–5 mg/dL)	↑ slight (≈ +1–3 mg/dL)	↓ slight (≈ −5 to −10 mg/dL)	↓ small-dense LDL; ↑ HDL2 (signal from limited cohorts)	Strong HF benefit; LDL-C rise generally small and manageable with statins
Dapagliflozin (10 mg)	↑ minimal to slight (≈ 0 to +3 mg/dL)	↑ (≈ +2 mg/dL)	↓ (≈ −10 mg/dL)	↓ small-dense LDL; shift toward larger buoyant LDL	More consistent TG reduction signal; beneficial metabolic effects
Canagliflozin (100–300 mg)	↑ small–moderate (≈ +3–7 mg/dL)	↑ (≈ +2 mg/dL)	↓ (≈ −5 to −10 mg/dL)	Limited data on particle remodeling	Most notable LDL-C increase in the class; monitoring ApoB advisable
Ertugliflozin / Others	↑ slight	↑ slight	↓ slight	Evidence insufficient	Limited HF-focused lipid data; likely similar class profile

In most studies, the magnitude of LDL-C increase ranges from 2-7 mg/dL, HDL-C increases by 1-3 mg/dL, and triglycerides decrease by approximately 5-10 mg/dL. These effects appear independent of glycemic status and consistent across both heart failure with reduced ejection fraction (HFrEF) and heart failure with preserved ejection fraction (HFpEF) populations [3-7]. Importantly, none of the major cardiovascular or heart failure outcome trials demonstrated an increased risk of atherosclerotic events attributable to these lipid changes [5,18,19].

Overall, the data indicate that the lipid profile modifications induced by SGLT2 inhibition are numerically small, mechanistically coherent, and clinically neutral in the HF population.

Drug-specific evidence in heart failure

Empagliflozin

Empagliflozin is associated with mild LDL-C and HDL-C increases and modest triglyceride reduction in both diabetic and non-diabetic patients [12,20-22]. Particle-level analyses demonstrate decreases in sd-LDL and improved HDL2 concentrations [12]. Given the robust HF benefits reported in EMPA-REG OUTCOME and EMPEROR trials [4-6], these minor lipid changes are unlikely to offset its overall cardioprotective effect.

Dapagliflozin

Dapagliflozin shows consistent triglyceride reduction and HDL-C elevation, with negligible LDL-C change [12,13,23-27]. Japanese cohort data confirm favorable reductions in sd-LDL and elevations in HDL2 [12]. Its beneficial effects on metabolic flexibility and endothelial function further support an anti-atherogenic metabolic profile [28].

Canagliflozin

Canagliflozin produces the largest LDL-C rise among SGLT2 inhibitors [18,19,21,29, 30], yet without an increase in cardiovascular events in long-term trials [18]. Evidence suggests that concurrent reductions in sd-LDL and triglycerides, coupled with improved cardiac energetics, may mitigate any theoretical atherogenic risk [19,21,29,30].

Emerging data also suggest the potential benefit of SGLT2 inhibitors in transthyretin amyloid cardiomyopathy. A recent cohort study [31] reported improvements in functional capacity and stabilization of cardiac biomarkers in patients receiving SGLT2 inhibitors, supporting the hypothesis that enhanced myocardial metabolic efficiency and improved substrate utilization may extend to infiltrative cardiomyopathies. While preliminary, these observations highlight an additional potential therapeutic domain for SGLT2 inhibition.

Clinical integration and practice considerations

From a clinical standpoint, SGLT2 inhibitors should not be regarded as lipid-modifying agents but rather as metabolic modulators with cardioprotective properties. In HF patients, observed lipid changes with SGLT2 inhibitors are generally modest and not associated with increased atherosclerotic or ischemic events. Across major outcome trials and HF-focused analyses, the small rises in LDL-C and HDL-C and mild reductions in triglycerides did not translate into higher ASCVD risk, and cardiovascular benefits were preserved irrespective of baseline lipid profile [5,12-14,20,21,32].

For individuals exhibiting mild LDL-C or ApoB elevations, standard lipid-lowering therapy with statins, possibly in combination with ezetimibe, remains the first-line strategy [32]. For patients with persistent LDL-C or ApoB elevations despite statin-ezetimibe therapy, or for those with statin intolerance, additional lipid-lowering options should be considered. Proprotein convertase subtilisin/kexin type 9​​​​​​​ (PCSK9) inhibitors (alirocumab, evolocumab) provide substantial reductions in ApoB and LDL-C and are appropriate for high-risk HF patients with residual atherosclerotic risk. Bempedoic acid offers an oral alternative with demonstrated LDL-C lowering, particularly useful in statin-intolerant individuals. Inclisiran, a small interfering RNA targeting hepatic PCSK9 synthesis, provides durable LDL-C reduction with twice-yearly dosing and may be advantageous in patients with limited medication adherence. These agents can be safely combined with SGLT2 inhibitors when indicated. SGLT2 inhibitors should be continued for their proven cardiorenal benefits, as discontinuation based on small lipid fluctuations is not supported by current evidence [21,32].

Thus, the lipid effects of SGLT2 inhibitors in HF should be interpreted within the broader context of their favorable metabolic remodeling and strong outcome benefits, rather than isolated biochemical variations.

Beyond biological and pharmacologic considerations, access to SGLT2 inhibitors in real-world HF care is strongly influenced by social determinants of health. Socioeconomic disadvantage, limited insurance coverage, and poverty have been associated with a lower likelihood of receiving guideline-directed medical therapy, including SGLT2 inhibitors, despite clear clinical indications. Recent population-level data demonstrate that patients from lower-income or structurally marginalized backgrounds face significant treatment gaps, underscoring the need for health-system and policy-level strategies to ensure equitable implementation of SGLT2 inhibitor therapy in HF [33].

Review

Sodium-glucose cotransporter 2 inhibitors have emerged as cornerstone therapy in heart failure (HF), consistently reducing HF hospitalizations and mortality independent of glycemic control [3-7]. However, their influence on lipid metabolism remains debated, particularly given the well-established role of dyslipidemia and lipotoxicity in HF pathophysiology [2]. In this narrative synthesis, we identified a pattern of modest quantitative lipid changes accompanied by potential qualitative improvements in lipoprotein composition, together suggesting a metabolic shift that may be neutral or even favorable in the HF population.

Across trials and observational studies, SGLT2 inhibitors induce minor increases in LDL-C and HDL-C and slight reductions in triglycerides [12,13,15,23]. While these changes are small in absolute magnitude, emerging data indicate reductions in small-dense LDL particles and increases in large buoyant HDL2 subfractions, particularly with dapagliflozin and empagliflozin [12,15]. Such remodeling patterns are metabolically advantageous, as sd-LDL and dysfunctional HDL phenotypes demonstrate stronger associations with adverse cardiovascular (CV) events than traditional lipid measurements [2]. Although particle-level evidence remains limited, it offers a mechanistic explanation for the lack of increased atherosclerotic risk despite small rises in LDL-C observed in EMPA-REG OUTCOME and CANVAS [5,19,34].

Mechanistically, SGLT2 inhibitors promote adipose lipolysis, hepatic fatty-acid β-oxidation, and ketogenesis, facilitating a shift toward more oxygen-efficient cardiac substrate utilization [8-11]. This "thrifty substrate" hypothesis suggests that enhanced ketone availability may improve myocardial energetics and reduce toxic lipid intermediates such as diacylglycerol and ceramides [8,10]. Concurrently, increased hepatic cholesterol flux may transiently expand intracellular cholesterol pools, reducing LDL receptor expression and producing the modest LDL-C elevation reported in metabolic studies [9,16]. Thus, lipid modifications may reflect redistributed substrate handling and lipotoxicity mitigation rather than pro-atherogenic signaling.

From a clinical standpoint, these subtle biochemical shifts should be interpreted within the broader context of robust reductions in HF events and mortality consistently observed across SGLT2 inhibitor trials [3-7,19,21,29,30,32,35-49]. Importantly, the slight LDL-C rise seen with certain agents, particularly canagliflozin, has not translated into excess ischemic events [18,45]. Dyslipidemia in HF should continue to be managed according to ApoB-driven, guideline-directed lipid-lowering strategies, including statins and ezetimibe when indicated, while maintaining SGLT2 inhibitors for their established cardiorenal benefit [21,32,34,39-43].

Heterogeneity in reported lipid effects likely reflects differences in HF phenotype (HFrEF vs HFpEF), baseline metabolic status, background statin therapy, trial duration, and lipid measurement methodologies. To date, no randomized trial has prospectively evaluated ApoB, LDL-particle number, sd-LDL, or HDL efflux capacity as primary endpoints in HF patients receiving SGLT2 inhibitors, representing a major knowledge gap. Future studies incorporating lipidomics, metabolomics, and advanced lipoprotein phenotyping are required to resolve mechanistic uncertainties and clarify the clinical significance of these biochemical adaptations [12,15,50,51].

In summary, SGLT2 inhibitors induce modest but mechanistically coherent lipid changes, characterized by slight increases in LDL-C and HDL-C and reductions in triglycerides, alongside possible improvements in lipoprotein quality. These effects occur in parallel with profound clinical benefits in HF and do not appear to confer adverse atherogenic risk. Collectively, available data support continued use of SGLT2 inhibitors as foundational HF therapy while acknowledging the need for HF-specific lipid outcome studies incorporating particle-level metrics.

## Conclusions

SGLT2 inhibitors consistently improve clinical outcomes in heart failure, and their effects on lipid metabolism - although modest in magnitude - appear metabolically coherent and aligned with improved substrate utilization, reduced lipotoxicity, and qualitative lipoprotein remodeling. A unique contribution of this review is the integration of quantitative lipid changes with emerging particle-level data and HF-specific metabolic pathways, offering a unified framework that has been largely absent from prior SGLT2i literature.

Future research should prioritize HF populations with high residual metabolic risk, including patients with HFrEF and coexisting diabetes, HFpEF with obesity or metabolic syndrome, and individuals with persistently elevated ApoB or triglyceride-rich lipoproteins despite guideline-directed therapy. Dedicated HF trials powered for lipid-centric primary endpoints - such as ApoB, small-dense LDL, LDL particle number, HDL functionality, and lipidomic profiling - are needed to clarify the clinical significance of these adaptations.

From a practical standpoint, SGLT2 inhibitors should be used for their evidence-based HF benefits regardless of small LDL-C changes, with guideline-directed lipid-lowering therapy optimized in parallel to achieve ApoB- and LDL-C-based targets.

## Figures and Tables

**Figure 1:**
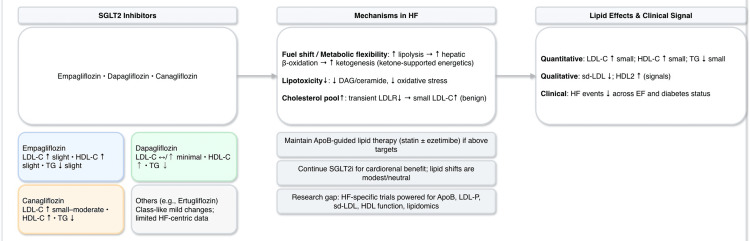
Graphical Abstract Source: Created by the authors. Abbreviations: SGLT2 = sodium–glucose cotransporter 2; HF = heart failure; LDL-C = low-density lipoprotein cholesterol; HDL-C = high-density lipoprotein cholesterol; TG = triglycerides; sd-LDL = small dense low-density lipoprotein; HDL2 = large buoyant high-density lipoprotein subfraction; ApoB = apolipoprotein B; LDLR = low-density lipoprotein receptor; DAG = diacylglycerol; RAAS = renin–angiotensin–aldosterone system; EF = ejection fraction

## Appendices

Appendix 1: Graphical abstract
